# EZH2 as a Regulator of CD8+ T Cell Fate and Function

**DOI:** 10.3389/fimmu.2020.593203

**Published:** 2020-10-06

**Authors:** Christopher J. Stairiker, Graham D. Thomas, Shahram Salek-Ardakani

**Affiliations:** Cancer Immunology Discovery, Worldwide Research, Development and Medical, Pfizer Inc., San Diego, CA, United States

**Keywords:** EZH2, enhancer of zeste homolog 2, CD8 lymphocytes +, effector, memory, cell fate and differentiation, virus, cancer

## Abstract

Enhancer of zeste 2 (EZH2) is the catalytic subunit of the Polycomb Repressive Complex 2 (PRC2) that mediates di- and trimethylation of histone 3 lysine 27 effectively precluding successful gene transcription at these loci. This class of epigenetic modifications facilitates the maintenance of tissue-specific cellular transcriptional programs as cells undergoing successive rounds of proliferation. CD8+ T cells are effective mediators of adaptive immunity and function to eliminate virus- and bacteria-infected cells as well as tumor cells. Upon recognition of cognate antigen, T cells undergo activation/proliferation to clear the target cells. The heterogeneous population of responding T cells formed during these proliferative events thus rely on epigenetic modifications to ensure identity and confer functional capabilities. In this review, we will focus on the role of the dynamic expression EZH2 in shaping the epigenetic landscape of CD8+ T cell fate and function, with a particular emphasis on infection and cancer. We also explore competing hypotheses pertaining to EZH2 function and the prospects of clinical EZH2 inhibitors in fine-tuning T cell responses.

## Introduction

**Figure 1:**
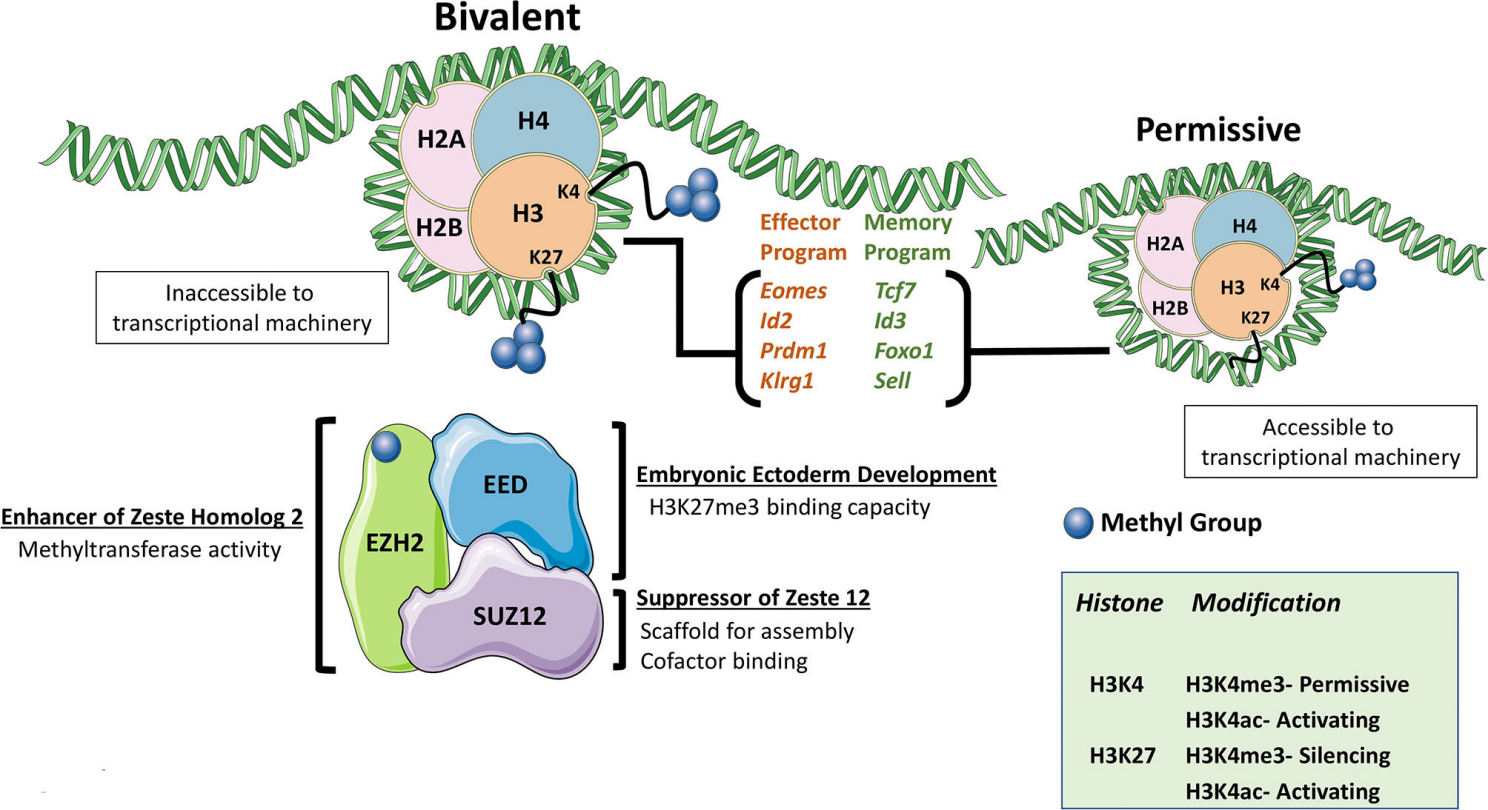
The PRC2 Complex. The PRC2 complex is formed mainly by EZH2, EED, and SUZ12. The PRC2 complex is responsible for the di- and trimethylation of H3K27 and makes the genomic DNA associated with chromatin inaccessible. Combination marks of H3K27me3 and H3K4me3 denote bivalent histones whereby removal of the repressive H3K27me3 mark allows for rapid gene induction as the H3K4me3 mark is permissive. When EZH2 function was lost, some of the most commonly cited differentially expressed gene are listed.

The Polycomb Repressive Complexes (PRCs) are large protein multimers that modify lysine residues on histones. The two primary PRCs noted in mammals are PRC1, formed by several distinct proteins but most notably RING1A/B and BMI1, and PRC2. The core subunits of PRC2 are enhancer of zeste homolog 1/2 (EZH1/EZH2), embryonic ectoderm development (EED), and suppressor of zeste 12 (SUZ12) (1). Within the PRC2 complex, EZH2 (and to a lesser extent, EZH1) is the catalytic subunit responsible for methyltransferase activity. Both EZH proteins were observed to be phosphorylated, although EZH1 phosphorylation was shown to lead to degradation, while EZH2 phosphorylation led to reduced function (2, 3). PRC2 mediates di- and trimethylation of histone 3 lysine 27 (H3K27), which is classified as a transcriptionally repressive mark while acetylation at this same residue is regarded as permissive for active transcription, making H3K27 post-translational modification status an important regulator of the cellular transcriptome (4). Histone marks can be broadly categorized as permissive or repressive based upon the effect it has for allowing or denying (respectively) successful gene transcription to take place at genomic DNA loci. Similar to H3K27 acetylation, other activation marks like H3K4 trimethylation (H3K4me3), are also associated with combinatorial histone modification required for permissive loci (5). Posttranscriptional modifications, namely differential splicing, have also been noted to be a function of these histone modifications, but remains far less explored (6, 7). Interestingly both H3K27 trimethylation (H3K27me3) and H3K4me3 marks can be present at single “bivalent” loci whereby removal of repressive H3K27me3 allows for rapid gene induction (Figure 1). PRC2-mediated inhibition of target-gene transcription via the repressive H3K27me3 mark is critical for lineage commitment and maintenance of cell identity (4, 8–10).

Upon recognition of cognate antigen bound to major histocompatibility (MHC) molecules displayed on the surface of antigen-presenting cells (APCs), T cells proliferate, differentiate, and exert functions (largely classed as effector, helper, or regulatory dependent on the cell type) in the lymph node or at peripheral sites. During acute infection, pathogen-infected cells are cleared by effector T cells, leaving behind a pool of memory T cells to protect against future re-infection. The differentiation of T cells is heavily influenced by the microenvironment, antigen persistence, and co-stimulatory signals that accompany T cell receptor (TCR) engagement, all of which impact the subsequent transcriptional and epigenetic profiles of the responding T cells (11). In contrast to acute resolving infections, studies performed with lymphocytic choriomeningitis virus (LCMV) strain clone 13, which establishes a prolonged infection, demonstrated that virus-specific CD8+ T cells progressively lose effector functions and accrue inhibitory receptors, defining an alternative T cell dysfunctional state known as exhaustion (12, 13). During chronic antigen stimulation, a heterogeneous pool of exhausted cells develops with distinct subpopulations demonstrating varying degrees of functionality and proliferative potential (14). The ability of exhausted T cells to proliferate upon PD-1/PD-L1 intervention is one of the most notable discoveries that differentiated exhausted cell subsets (15). Assay for transposase-accessible chromatin using sequencing (ATAC-Seq) of these subpopulations suggested relatively unique chromatin landscapes (compared to effector, naïve, and memory cells), suggesting exhausted T cells possess a unique epigenetic program (16–18). However, the role(s) of different chromatin-modifying enzymes in the establishment of this epigenetic state remains unclear.

The PRC2 complex, specifically EZH2, acts to effectively integrate the environmental stimuli received by individual T cells during activation and proliferation, thereby shaping the downstream epigenetic landscape of the response at a population level. Many studies have focused on the role of EZH2 in different CD4+ T helper (T_H_) cell fates, including lineage-defining transcription factor expression and cytokine production, covered elsewhere (19–22). The purpose of this review is to instead focus on the role of EZH2 in CD8+ T cell differentiation and effector function in the context of viral infection and cancer, comparing and contrasting phenotypes observed across multiple models to give greater insight into EZH2 biology.

## H3K27me3 in CD8+ T Cell Differentiation

One of the first studies to characterize the unique epigenetic landscape of different human CD8+ T cell subsets was achieved by chromatin immunoprecipitation sequencing (ChIP-Seq) and microarrays on stimulated human enriched peripheral blood CD8+ T cells sorted based on markers for naïve (CD62L+CD45RA+), central (CD62L+CD45RA-), or effector (CD62L-CD45RA-) memory cells (23). By comparing the histone methylation profiles of these distinct subpopulations, it was shown that the number of genes with H3K27me3 deposition was constant across populations. However, differences in the density distribution of H3K27me3 at sites between cell fates were not addressed (23). Unlike H3K27me3, the number of detected genes acquiring H3K4me3 demonstrated an increasing trend from naïve to central memory and effector memory cells, suggesting a greater reliance on this modification in determining T cell subpopulation transcriptional dynamics (23). These authors identified active (H3K4me3 rich), repressed (H3K27me3 rich), and bivalent nucleosomes, found to possess concurrent H3K27me3 and H3K4me3. Bivalent loci were detected in both central and effector memory subsets with few overlapping genes. However, considerable overlap might be expected, the low number of shared genes may reflect the limited breadth of transcripts detected by microarray (23). Notably, combining these histone profiles with the matched data generated from microarrays, bivalent loci were observed to permit the rapid de-repression of genes by memory cells upon activation to facilitate accelerated target gene transcription, conferring the memory compartment with the ability to rapidly respond to cognate antigen upon re-encounter (23). Similar findings in terms of relative histone modification distribution were corroborated in studies utilizing adoptive transfer of CD8+ T cells expressing the transgenic TCR (Vα1Vβ5) specific for ovalbumin (OVA_257−264_) (OT-I). Upon infection with a recombinant influenza virus (A/HKx31-OVA) expressing OVA, naïve, effector, and memory cells were profiled for H3K4me3 and H3K27me3 histone marks. The presence of bivalent marks was observed in naïve cells at major transcription factor loci that were transcriptionally upregulated upon activation, including *Eomes, Tbx21*, and *Irf4* (24). Consistent with observations made of the epigenetic marks found in subsets of CD4+ T cells, H3K27me3 was primarily found in intergenic regions, accounting for 60-70% of methyl group deposition and found to be relatively consistent across naïve, effector and memory populations. In contrast, promoter and other genomic loci accounted for a much smaller fraction across all CD8+ T cell subsets (23–25). Genes sets were batched into modules that were shared or exclusive to one or two of the sorted populations (24). For example, at the genes *Sell, Bcl2, Bcl11b, Gzma*, and *Gzmk*, high H3K4me3 and low H3K27me3 were present in naïve and memory OT-I's, however, H3K27me3 was higher in effectors at these loci (24). These CD8+ T cell profiles reflect the importance of H3K27me3 (and H3K4me3) in regulating gene expression and, ultimately, T cell function and identity. In order to determine the more direct role of EZH2, other studies utilizing conditional deletion and pharmacological inhibition were performed as described in later sections.

## Regulation of CD8+ T Cells During Infection

The Armstrong strain of LCMV produces an acute infection lasting ~7–10 days and generates a robust, effector T cell population. During the expansion phase, antigen-specific cells can be broadly categorized as a short-lived effector cell (SLEC) or memory precursor effector cell (MPEC) (26). SLECs, phenotyped as KLRG1+CD127–, have low memory potential and overt effector capacity compared to the MPEC counterpart, identified as CD127+KLRG1–, characterized by the greater potential to seed memory CD8+ T cell pools and reduced ability to exert effector functions (26). This model also lends itself to understanding T cell-intrinsic properties using adoptive transfer of CD8+ T cells expressing a transgenic TCR (Vα2Vβ8) recognizing the LCMV glycoprotein (GP_33−41_) (P14). Kakaradov and colleagues performed single-cell RNA sequencing (scRNA-Seq) of P14 T cells isolated at different time points after infection with LCMV Armstrong to profile the heterogenous antigen-specific CD8+ T cell population, taking advantage of the P14 system to normalize the effect of TCR affinity. They identified EZH2 as preferentially expressed by the effector like progenitor population rather than the memory progenitor cells (27). To examine the T cell-intrinsic effects of *Ezh2* deletion, *Ezh2*^*fl*/*fl*^*Cd4*^*Cre*^ P14 T cells were adoptively transferred into wildtype recipient mice and subsequently infected with LCMV Armstrong. Initial activation and proliferative potential were intact in *Ezh2*-floxed CD8+ T cells; however, during later time points, there was reduced recovery of donor *Ezh2*^*fl*/*fl*^*Cd4*^*Cre*^ P14 T cells compared to wildtype donors accompanied by reduced cytokine production upon stimulation (27). ChIP-Seq of wildtype CD8+ T cells confirmed the dependence of the effector program by identifying memory associated genes as EZH2 targets, including *Tcf7, Eomes, Smad2, Bcat, Opa1, Klf2, Id3*, and *Foxo1*, suggesting that repression of the memory program by EZH2 was necessary for successful effector generation (27).

By profiling the H3K27me3 and H3K27 acetylation (H3K27ac) status of P14 SLECs and MPECs at day 10 post-infection, Gray et al. corroborated the importance of EZH2 for the effector program. Here, memory associated genes (*Id3, Tcf7, Bach2*, and *Bcl2*) were preferentially enriched for H3K27me3 marks in SLECs compared to MPECs (28). Another conditional deletion model was employed to delete *Ezh2* in recently activated cells by placing Cre-recombinase under the control of effector molecule granzyme B (28). When infected with LCMV Armstrong, these *Ezh2*^*fl*/*fl*^*Gzmb*^*Cre*^ mice demonstrated a similar phenotype to that observed by Kakaradov *et al*., with reduced antigen-specific cells despite similar initial activation and proliferation, reduced effector cytokine expression, and an enriched memory phenotype, suggesting a compromised effector program (27, 28). Furthermore, many of the transcription factor genes with reduced H3K27me3were confirmed to be upregulated at the protein level, including TCF1 (*Tcf7*), FOXO1, and Eomes (28). Using a GranzymeB-ER^T2^Cre inducible system, they also demonstrated that tamoxifen-mediated deletion of *Ezh2* during the acute response did not compromise the formation of memory cells. However, when low numbers of *Ezh2*^*fl*/*fl*^*Gzmb*^*Cre*^ GP_33−41_-specific memory CD8+ T cells were transferred into naïve hosts, they were unable to expand to the same extent as *Ezh2* sufficient memory counterparts and clear the infection as efficiently when challenged with recombinant *Listeria monocytogenes* engineered to express LCMV glycoprotein_33−41_ (27). To answer why memory associated genes are aberrantly expressed in effector cells, Gray et al. suggested that in the absence of EZH2 repressive marks, FOXO1 binding sites at memory precursor gene loci are exposed enabling access of transcriptional machinery to upregulate this genetic program which is normally inaccessible when EZH2 is present (28).

Despite the commonalities present with the LCMV model, a subsequent study utilizing recombinant vaccinia viruses (rVacV) demonstrated different results while verifying others. Using a rVacV expressing either OVA or gp100, He et al. phenotyped the responding T cells and observed that in this model, the SLEC population was enriched earlier during infection (29). Selective enrichment of MPEC's in the LCMV model (~day 8) and the enrichment of SLEC's here (~day 5) represent a potential difference in models. However, they may be reconciled by the different time points, replicative capacity of the pathogens, route of infection, or cytokine milieu. Among the genes differentially expressed by activated *Ezh2*^*fl*/*fl*^*Cd4*^*Cre*^ CD8+ T cells were *Id2, Id3, Eomes*, and *Prdm1*, maintaining the common theme of an aberrant memory-associated gene program induction (27–29). In line with the previous model, the memory recall response was impaired in terms of proliferation and effector cytokine production using an *in vivo* dendritic cell priming method for the initial activation followed by *ex vivo* Cre-mediated deletion of *Ezh2* and subsequent cognate antigenic stimulation (29). Interestingly, He *et al*. were able to abrogate many of the transcriptional differences observed in *Ezh2*^*fl*/*fl*^*Cd4*^*Cre*^ CD8+ T cells by expressing a constitutively active phosphorylation insensitive mutant EZH2. The functionality of this EZH2 variant was confirmed by greater H3K27me3 marks than either the vector control or EZH2 rescued CD8+ T cells, and this restored some of the lost transcriptional programming observed in *Ezh2*^*fl*/*fl*^*Cd4*^*Cre*^ T cells and increased the recovery of effectors (29).

Infection with *Listeria monocytogenes* also bore commonalities with the LCMV and VacV models. Chen *et al*. observed a greater skewing toward SLEC rather than MPEC cells and greater functionality in terms of IFNγ, IL-2, and granzyme B production upon *in vitro* activation. During the acute response, *Ezh2*^*fl*/*fl*^*Cd4*^*Cre*^ CD8+ effector T cells failed to persist as well as wildtype counterparts *in vitro* and *in vivo* (30). Despite this, the use of the activation-induced deletion model (*Ezh2*^*fl*/*fl*^*Gzmb*^*Cre*^) presented different results with no observed increase in apoptosis, yet reduced cell frequencies of *Ezh2*-deficient cells. Citing the altered expression in cell cycle-related genes *Cdkn2a* and *Cdkn1c*, the authors proposed that *Ezh2*^*fl*/*fl*^*Gzmb*^*Cre*^ CD8+ T cells had slower doubling time, which ultimately resulted in reduced antigen-specific cell numbers (30). Notably, Gray *et al*., using the same model, demonstrated equal doubling times *in vitro* and *in vivo* (28). These phenotypic differences may be attributable to other factors such as TCR affinity or differential requirement for costimulation (28, 30). It is interesting to speculate that this slowed doubling time proposed by Chen *et al*. might be due to a previously identified role of EZH2 in the DNA damage response. In a separate study, EZH2 was found to co-precipitate with Ku80, a DNA damage response protein, and phosphorylated by DNA-dependent protein kinase, which inhibited methyltransferase activity (31). The study suggested that EZH2 inhibition resulted in greater DNA damage and apoptosis in activated T cells. This provides an alternative mechanism to the previous studies, suggesting that in pathogen responding T cells where DNA replication occurs rapidly, loss of EZH2 increases CD8+ T cell susceptibility to DNA-damage mediated apoptotic cell death (31).

**Figure 2:**
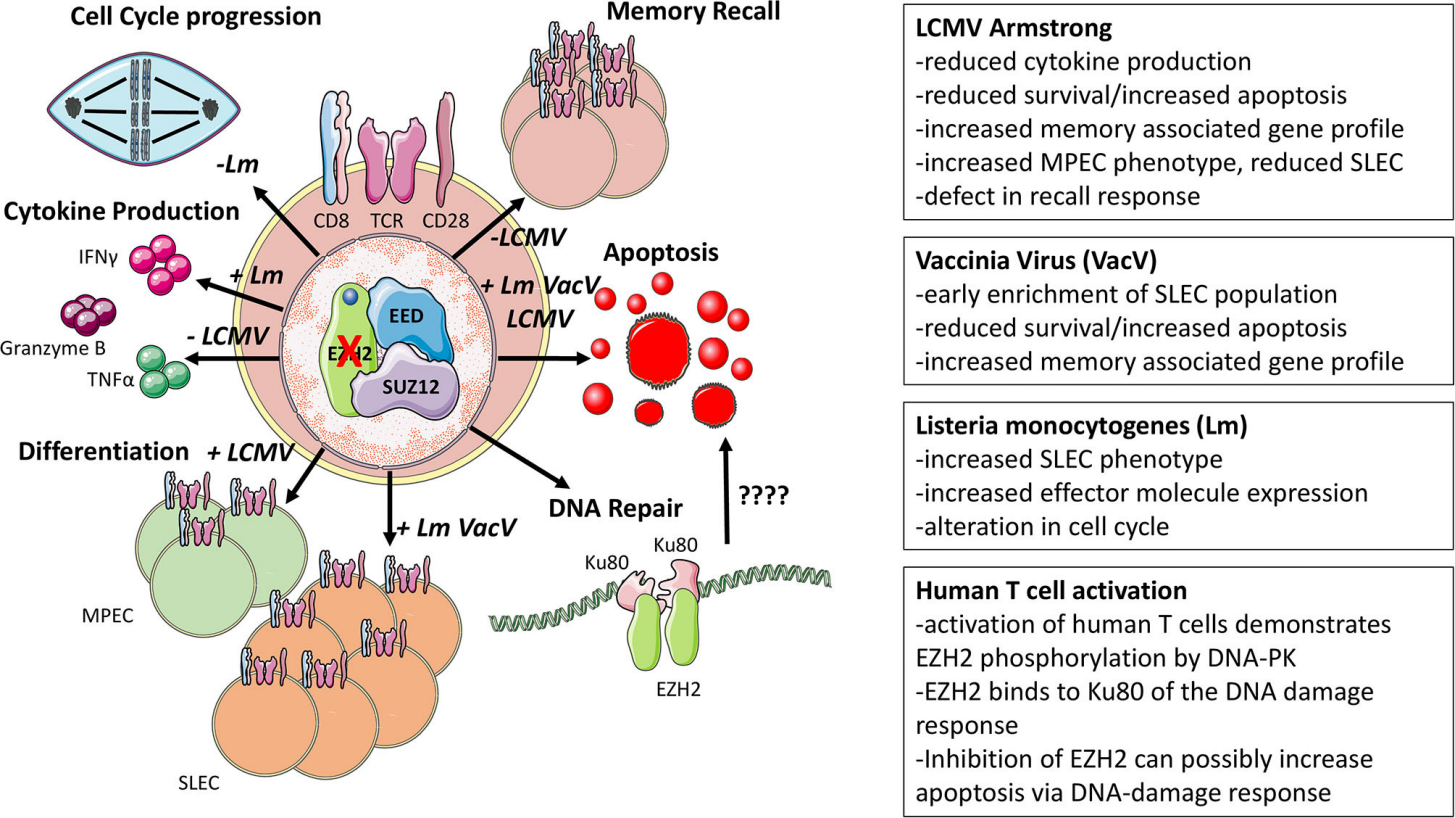
Infection-associated models of EZH2. Major phenotypes associated with modulating EZH2 function and mechanisms of EZH2 function in models of infection are displayed. Distinguishing features found in each model are denoted by abbreviations of the pathogen used in each setting.

The role of EZH2 in memory cells is relatively consistent across temporal deletion models. Both the LCMV model and re-stimulation of *in vivo* primed cells suggest that *Ezh2*-deficient T cells are unable to produce effective recall responses in terms of cytokine production and the ability to clear invading pathogens (28, 29). Complete ablation models of EZH2 mainly using *Ezh2*^*fl*/*fl*^*Cd4*^*Cre*^ mice may exert somewhat different effects than pharmacological inhibition, for instance, genetic deletion ablates EZH2 expression, potentially allowing EZH1 to compensate within the PRC2 complex. Furthermore, the timing of EZH2 inhibitor administration, e.g., during activation, differentiation, contraction, restimulation, etc., may very well impact the T cell phenotypic and functional response. This poses a different question of whether the duration of EZH2 inhibition could yield different effects on T cells than those presented in Cre-mediated deletion models where the effect is more absolute. EZH2 inhibitor studies also bear more translational relevance than *Ezh2* deletion models. Indeed, profiling of transcripts during the effector cell program suggests a more complex and phasic transcriptional dynamic of gene induction than a simplistic on- or off-model (27). Furthermore, to what extent EZH1 may compensate for loss of EZH2 in these models is not addressed, perhaps due in part to the segregated expression patterns where EZH2 is expressed more in actively dividing cells and EZH1 more in differentiated populations (4). Considering the fact that there is some overlap between memory and naïve gene programs, it could be possible that in the absence of EZH2, EZH1 occupies the PRC2 complex. EZH1 occupancy in the PRC2 complex then may theoretically result in not a memory phenotype but sustaining portions of the naïve program. From infection models, differentiation, effector function, survival, and memory recall are few of the processes affected by modulating EZH2 activity, with some effects being pathogen dependent (Figure 2). This leaves more open questions about the different effects of EZH2 inhibition *in vivo*.

## Regulation of CD8+ T Cells During Cancer

*Ezh2* is negatively affected by factors present in the tumor microenvironment (TME) as evidenced by the reduction of *Ezh2* transcription when T cells were activated in the presence of lyophilized tumor supernatant (32, 33). Notably, one study observed that human CD8+EZH2+ T cells did not express KLRG1, TIM-3, or CD57, suggesting that EZH2 is primarily expressed in cycling or activated cells, not in senescent or anergic cells (15, 33, 34). With the advent of ATAC-Seq, many have profiled the epigenetic landscapes of naïve, effector, memory, and exhausted human and murine T cells in the context of cancer and chronic infection with the consensus being that exhaustion represents an alternative T cell fate whose chromatin landscape is quite disparate from the other T cell states (16, 17, 35, 36). Differences in chromatin accessibility beg the question of what role chromatin-modifying proteins like EZH2 play in the formation of these epigenetic landscapes and if it can be manipulated. However, little is known about EZH2-mediated chromatin modification in the context of chronic antigen stimulation.

Phenotypic analysis of human CD8+EZH2+ cells suggested that this population had greater effector capacity and reduced sensitivity to apoptosis (33). CD8+EZH2+ T cells from healthy donors and ovarian cancer patients were polyfunctional, defined by their capability to coproduce IFNγ, TNFα, and granzyme B, and resistant to apoptosis, in part due to their elevated Bcl-2 expression (32, 33). Consistent with this, inhibition of EZH2 via pharmacological inhibitors (DZNep or GSK126) or RNA interference (RNAi) reduced the frequency of polyfunctional (triple positive) human CD8+ T cells and increased the frequency of apoptotic cells *in vitro* (33). Although DZNep treatment has been shown to inhibit effector CD8+ T cell function, an interpretational caveat is advised as this drug broadly inhibits methyltransferases and is known to have non-EZH2 mediated effects in cancer cells (37, 38). Nevertheless, this loss of polyfunctionality and viability was attributed to the inability of EZH2 to suppress inhibitors of NOTCH signaling (specifically *Numb* and *Fbxw7*), which is essential for effector differentiation (33, 39). As to what causes the decrease in EZH2 function, Zhao *et al*. suggested that glucose restriction induces microRNAs targeting *Ezh2* (33). Using a similar model, in which T cells are cultured in lyophilized conditioned media from tumor cells, Long et al. recapitulated the increase in microRNA-26 to corroborate Zhao *et al*.'s findings. However, they suggested that unknown soluble factors within the TME mediate the induction of inhibitory microRNA expression (32, 33). Using a humanized ovarian cancer model, DZNep-treated T cells were unable to control tumor metastasis to the same extent as control counterparts (33); similarly, CD8+ T cells transduced with a microRNA-26 decoy reduced tumor growth rate in an adoptive transfer melanoma model (32). As effector CD8+ T cells are reliant on glucose, it is tempting to speculate that while Akt signaling induces glucose receptor expression, without substrate in the glucose-poor TME, these inhibitory microRNA's are induced to suppress T cell function via EZH2 inhibition (40). This is supported by the role of increased Akt signaling favoring effector cell differentiation (41).

Furthermore, glucose restriction is known to reduce cytokine production, like IFNγ, producing a link between the loss of EZH2 and reduced effector function (42). Antitumor immune responses are known to be impaired upon loss of NOTCH2 signaling as NOTCH2-deficient mice exhibited increased tumor growth rates (43). Being that EZH2 inhibited CD8+ T cells are proposed to have defective NOTCH signaling in cancer, this theory could very well explain the reduced SLEC phenotype observed in the LCMV model as, during viral infection, NOTCH signaling is important for effector cell generation, particularly during influenza virus challenge (27, 28, 33, 39).

To understand the CD8+ T cell-intrinsic role of EZH2 in the antitumoral immune response, an adoptive transfer model was exploited, utilizing the B16 melanoma model in conjunction with T cells bearing a transgenic TCR (Vα1Vβ13) recognizing the melanoma glycoprotein gp100_25−33_ (Pmel) (44). He et al. transferred *Ezh2*^*fl*/*fl*^*Cd4*^*Cre*^ Pmel CD8+ T cells into melanoma B16-tumor-bearing mice, and showed that these cells were incapable of mediating the same tumor growth inhibition as *Ezh2*-sufficient cells (29). Upon transfer of *Ezh2*^*fl*/*fl*^*Cd4*^*Cre*^ CD8+ T cell into recipient animals and subsequent immunization with peptide-pulsed dendritic cells, T cells lacking *Ezh2* were recovered at lower numbers than control cells and accompanied by reduced IFNγ production, similar to the observations in infection models (28, 29). Citing the fact that the EZH2 function is controlled by phosphorylation, they were able to rescue many of the defects observed in *Ezh2*^*fl*/*fl*^*Cd4*^*Cre*^ CD8+ T cells by incorporating a phosphorylation insensitive EZH2 or pharmacologically inhibiting the upstream kinase (Akt) (2, 29). Previous work employing a *Listeria monocytogenes* infection model to understand the relative contribution of *Id2* and *Id3* to SLEC vs. MPEC differentiation had supported a role for *Id3* in promoting long lived memory cells (45). Transcriptionally, Id3^Hi^ cells expressed more memory-associated genes such as *Il7r, Sell*, and *Bcl2* while maintaining lower levels of effector-associated genes like *Gzmb* and *Prdm1* (45). Mechanistically, He *et al*. proposed a model whereby EZH2 functions to induce Id3, promoting memory cell formation and loss of EZH2 leads to augmented effector genes, including *Id2, Eomes*, and *Prdm1* which compromises the longevity of the antitumor response (29). Although typically associated with memory differentiation, this increased induction of *Eomes* is likely part of the general early effector program of recently activated CD8+ T cells (46, 47).

Common ground in prior work is found concerning EZH2 expression and function in metabolism centered around Akt signaling. Zhao *et al*. suggest that glucose restriction drives the inhibitor microRNA-mediated suppression of *Ezh2* expression. At the same time, He *et al*. argue that post-translational modification of EZH2 inhibits its function; both ultimately culminate in reduced EZH2 activity (29, 33). As Akt regulates EZH2 phosphorylation and is intimately linked with the metabolic function of T cells, EZH2 activity and expression can be viewed as a metabolic readout for CD8+ T cells (29, 33, 41). This provides a working model whereby restrictive glucose environments promote EZH2 phosphorylation and microRNA mediated *Ezh2* mRNA decay, limiting the function of EZH2. Thus, the expansion of effectors would theoretically correlate with an increase in the CD8+EZH2+ T cell population. This would explain why Zhao et al. observed that triple positive (IFNγ, TNFα, and granzyme B) T cells were enriched in the CD8+EZH2+ ovarian infiltrating TIL's and Goswami *et al*. noted that matched patient samples pre- and post-ipilimumab (anti-CTLA-4) treatment exhibited an increase in the frequency of CD8+EZH2+ T cells, theoretically, indicative of mobilization of effectors (33, 48).

Despite the previous publications, others have observed beneficial effects from inhibiting EZH2 activity. In the murine B16 melanoma model, Zingg et al. demonstrated that an EZH2 inhibitor (GSK503) combined with IL-2 complexed with an anti-IL-2 monoclonal antibody (NARA1) or anti-CTLA-4 therapy, reduced tumor growth (49). In a murine bladder model (MB49), Goswami *et al*. also showed that the combination therapy of EZH2 inhibition (via CPI-1205) and anti-CTLA-4 were capable of mediating a more significant tumor growth inhibition than the respective monotherapies (48). These antitumor effects were attributed to increased expression of genes associated with MHC-I peptide processing and presentation and increased expression of T cell-recruiting chemokines *Cxcl9* and *Cxcl10* (48, 50, 51). Thus, EZH2 is a relevant target in cancer therapies attempting to improve T cell recruitment into the TME. Specifically, expression of CXCR3 on T cells, which binds to ligands CXCL9/CXCL10, was highlighted in recent findings to be critical for drawing T cells into the TME and informs the design of future therapeutics with a primary aim of improving T cell recruitment (49, 52–54). Furthermore, the effect of EZH2 inhibition on destabilizing the regulatory T cell lineage is another primary consideration that was demonstrated to impact antitumor immunity (48, 55). In addition, both studies noted that EZH2 inhibition resulted in positive changes in the CD8+ T cell antitumor immune responses, including an increase in the frequency of IFNγ-producing CD8+ T cells and reduced inhibitory marker expression on tumor-infiltrating T cells (48, 49). These *in vivo* effects, however, were not suggested to be direct but rather an indirect effect of EZH2 inhibition on Treg function resulting in decreased CD8+ T cell suppression. From *in vitro* human CD4+ and CD8+ T cell cultures with tumor cell lines, incubation with CPI-1205 modestly increased cytotoxicity, but other direct effects on T cells were not demonstrated *in vivo* (48). Direct effects of EZH2 inhibition on the tumor itself are not likely to contribute to this phenotype as *Rag* knockout mice implanted with MB49 tumor cells and treated with an EZH2 inhibitor (CPI-1205) were observed to have equivalent survival and tumor growth kinetics as untreated controls (48). Supporting the future consideration of EZH2 inhibitors as immune-modulatory therapeutics is a published case study in which a metastatic chordoma patient was placed on a tazemetostat, an EZH2 inhibitor, regiment before receiving radiotherapy at the primary tumor site (56). Upon follow up, regression of metastatic tumor sites was observed (abscopal effect) along with increased immune cell infiltrates, noting an increase in proliferating (as marked by Ki-67 positivity) CD8+ T cells (56). Apart from human *ex vivo* studies and xenograft approaches, most studies assessing EZH2 manipulation in CD8+ T cells have relied on mouse models, which necessitates further corroboration in appropriate human systems to validate these findings.

**Figure 3:**
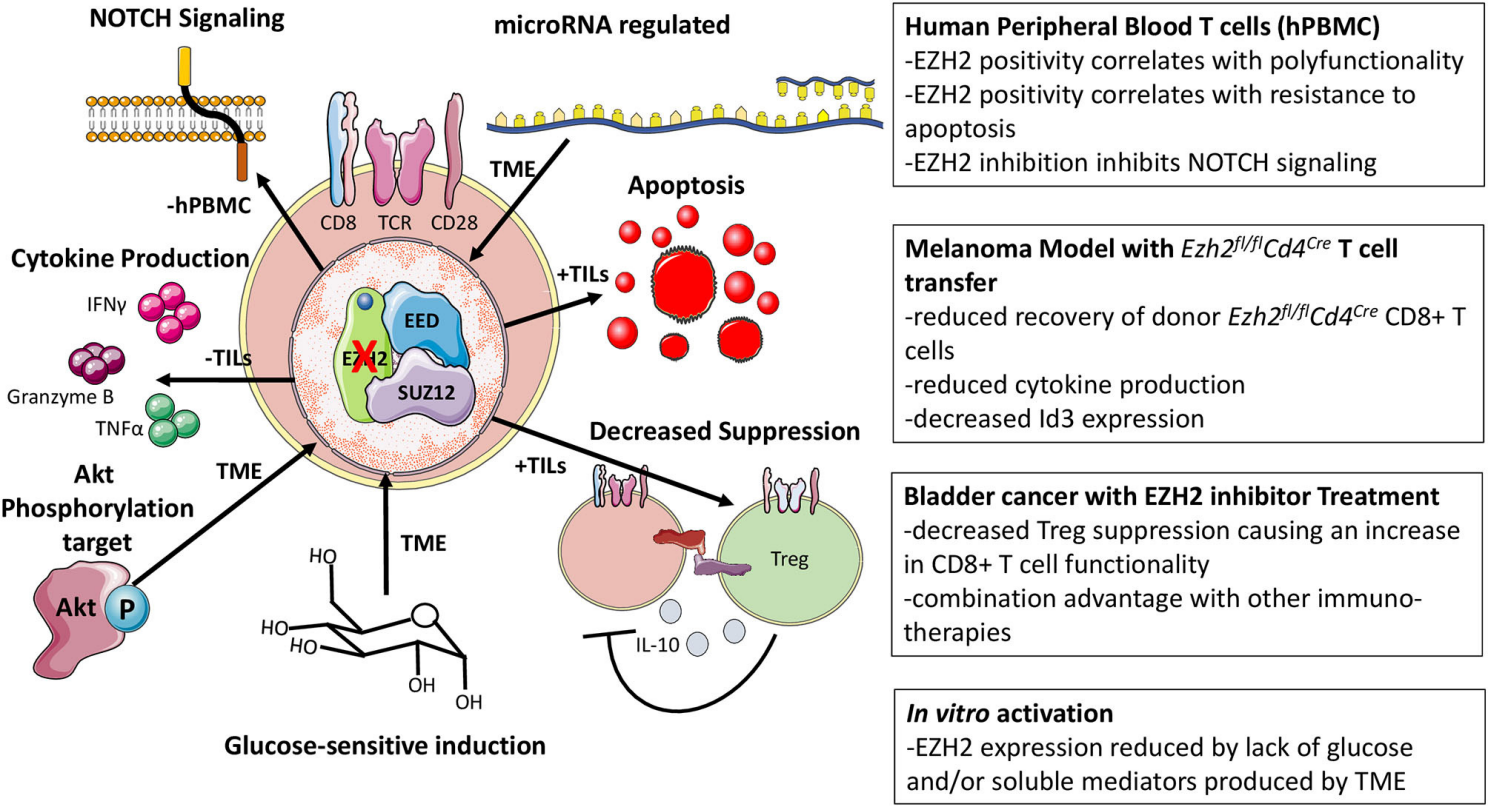
Cancer-associated models of EZH2. Observations associated with the loss of function EZH2 has on CD8+ T cells in cancer models as well as how EZH2 function and expression are modulated.

Global deletion of *Ezh2* in mature naïve T cells as in the *Ezh2*^*fl*/*fl*^*Cd4*^*Cre*^ T cells obscures the possible benefits of EZH2 inhibition, where potency of EZH2 inhibitors as well as pharmacological inhibitor treatment of the whole organism can alter the functional outcomes as observed in cancer models (33, 48). This difference in ablation of EZH2 vs. EZH2 inhibition may well explain why in some cancer models, EZH2 modulation can negatively or positively impact the antitumoral immune response. The same sort of potency must also be considered for the activation of CD8+ T cells *in vitro* with EZH2 inhibitors, where concentrations of compounds are likely not physiologically achieved *in vivo*. Thus, EZH2 inhibitor treatment of tumors in combination with immunotherapeutic agents should not be abandoned but may require careful consideration regarding timing and combination therapies in order to obtain the antitumoral benefits of EZH2 inhibition and spare or promote T cell function in the TME. From cancer models, the regulation of EZH2 expression and function is more fully elucidated and how its modulation in both CD4+ and CD8+ T cells can, directly and indirectly, impact the CD8+ T cells response (Figure 3).

## Discussion

From these studies, it is clear that *Ezh2* ablation often compromises the effector CD8+ T cell response. The mechanism by which they are compromised, however, remains controversial (Table 1). Some studies suggest that targeting EZH2 can confer higher sensitivity to apoptosis via either increased *Bim* or decreased *Bcl2* expression, reducing the number of effectors (27–29, 33). Others propose a model where doubling time is reduced (30). The phenotypic changes in *Ezh2-*deficient T cells were attributed to an altered transcription factor profile noting differences in *Id3, Id2, Eomes, Prdm1, Tcf7*, and *Foxo1* expression (27–29). These explanations are not mutually exclusive, and all could contribute to the phenotypic outcome of *Ezh2-*deficient or EZH2 inhibited CD8+ T cells. Acute pathogen infection models suggest that loss of *Ezh2* selectively compromises SLEC or MPEC populations, depending upon the model, yet agree that the responding acute CD8+ T cell population exhibits reduced survival and altered cytokine production. These differences in phenotypes and profiles may reflect differences in TCR affinity, activation environment, and antigen availability. However, another consideration is the speculated roles of EZH2 in TCR signaling supported by evidence of non-nuclear localization (57, 58). Although seemingly disparate, EZH2's proposed cytoplasmic involvement in TCR signaling could very well explain the differences in CD8+ T cell differentiation as TCR signal strength is known to influence memory versus effector cell fate (46, 59). In the context of cancer, there is chronic antigen stimulation where such phenotypes as SLEC and MPEC may no longer be applicable, making the comparison to acute pathogenic infection more complicated in defining the CD8+ T cell subpopulations affected by EZH2 inhibition.

**Table 1:** Key Findings from different EZH2 models.

**Model**	**Genotype**	**Phenotype**	**Associated genes/Proteins**	**References**
**Infection**				
L. monocytogenes-OVA	*Ezh2^*fl*/*fl*^Cd4^*Cre*^*	Reduced OVA-specific cells		(30)
	*Ezh2^*fl*/*fl*^Cd4^*Cre*^*-OT-I	Reduced Recovery; SLEC Skewed; Increased apoptosis		(30)
	*Ezh2^*fl*/*fl*^Gzmb^*Cre*^*	Reduced OVA-specific; no change in apoptosis; reduced cell cycling		(30)
LCMV	*Ezh2^*fl*/*fl*^Gzmb^*Cre*^*	Reduced effector cells; increased viral burden; normal initial expansion; reduced TNFα production; CD127+CD62L+CD27+KLRG1Lo	Increased: FOXO1, Eomes, TCF-1, CD62L, CD27; Decreased: Tbet and KLRG1	(28)
	*Ezh2^*fl*/*fl*^Cd4^*Cre*^*-P14	Reduced effector cells; reduced IFNγ and TNFα production; increased apoptotic cells; Increased CD25^Lo^CD62L^Hi^ cells		(27)
Vaccinia virus	*Ezh2^*fl*/*fl*^Cd4^*Cre*^*-Pmel-I	Reduced frequency at acute time point; decreased frequency of IFNγ producing cells		(29)
*In vitro activation*	*Ezh2^*fl*/*fl*^Cd4^*Cre*^*	Increased IFNγ, IL-2, Granzyme B; Increased apoptosis; Proliferation defects (CTV, BrdU)	Microarray: *Ifng; Cdkn2a, Cdkn2b, Cdkn1c*,	(30)
	*Ezh2^*fl*/*fl*^Gzmb^*Cre*^*	Increased cell cycling time	qRT-PCR: *Cdkn2aV1/V2*	(30)
**Cancer**				
	*Ezh2^*fl*/*fl*^Cd4^*Cre*^*-Pmel-I	Reduced donor frequency/number; reduced TGI		(29)
	Pmel-I transduced with phosphorylation insensitive EZH2	Increased frequency/number; Increased number of IFNγ producing cells	RT-PCR: Increased: Id3; decreased: *Prdm1, Eomes*	(29)
	EZH2 inhibited activated hPBMC isolated T cells	Reduced polyfunctionality (IFNγ, TNFα, Granzyme B); increased apoptosis; decreased NOTCH signaling	Increased: *Numb, Fbxw7;* Decreased: *Hes1, Hey1, Hey2*	(33)

*Summation of the different conditional deletion and inhibition experiments targeting EZH2 or modulated EZH2 in T cells. Included in the table are mRNA and protein targets that were noted to be affected by modulating EZH2 activity*.

Furthermore, proliferative events are not limited to the lymph nodes, as T cells are known to undergo a subsequent proliferation *in situ*, which could suggest differing effects of EZH2 inhibition on T cells during priming vs. post-activation proliferative bursts (60, 61). As an alternative T cell fate, T cell exhaustion is accompanied by many changes in chromatin accessibility (17, 35, 36). Moreover, the progression or phases of exhaustion are illustrated by defining transcriptional profiles suggestive of different chromatin landscapes (18). What role EZH2 and other chromatin-modifying complexes play in establishing these profiles is unclear. From a translational standpoint, more relevant studies using pharmacological inhibition of EZH2 are needed to understand the possible therapeutic potential and give greater insight into regulation of T cell responses by EZH2.

Using scRNA-Seq, the heterogeneity present within the CD8+ T cell population is more appreciated, even in chronic antigen stimulation, where more subpopulations of exhausted cells are defined (18). Extrapolating from the evidence of distinct RNA transcriptomes, this would suggest unique chromatin landscapes might be further appreciated using single cell ATAC-seq as well. Understanding the contribution of individual chromatin-modifying complexes to the regulation of these landscapes remains relatively unexplored. The different proliferative potential of subpopulations would imply differential responses to EZH2 inhibition, as proliferating cells are known to upregulate proteins like EZH2 to re-establish chromatin landscapes in daughter cells. Indeed, the role EZH2 plays in chronic antigen stimulation during viral infection is relatively unexplored as no known studies have utilized conditional deletion models in the LCMV Clone 13 model or pharmacologically inhibited EZH2 in human CD8+ T cells isolated from chronic virally infected patients. It remains to be determined which CD8+ T cell subpopulations are responsive to epigenetic modifying therapeutic intervention and at what stage their delivery would most effectively augment the antitumor or anti-pathogen immune responses.

One can then infer that there would be differential requirements for proteins like EZH2 in forming this epigenetic landscape, and thus inhibition of EZH2 could theoretically offer differing therapeutic benefits. In cases of chronic antigen stimulation like cancer and viral infections, the stem cell-like progenitor population identified by TCF-1 expression is associated with self-renewal, the ability to give rise to effectors, as well as responsiveness to PD-1 blockade (15). Indeed, acute viral models have shown that loss of *Ezh2* results in increased expression of memory-associated transcripts such as *Tcf7*, which is associated with stem-like CD8+ T cells in cancer (62). It is interesting to speculate that the proper timing of EZH2 inhibition could expand pools of stem-like CD8+ T cells, which could seed higher pools of effectors to increase antitumor and antiviral immunity (28, 63). Further studies are also required to establish putative therapeutic regimens that would capitalize on EZH2 inhibition in combination with immunotherapies targeting PD-1, CTLA-4, and others. For example, perhaps the use of EZH2 inhibitors before anti-PD-1 treatment could theoretically expand the pool of stem cell-like CD8+ T cells that could then be subsequently targeted by PD-1 blockade to enhance the effector response.

The control of EZH2 expression and induction also remains relatively unclear. One paper suggests that upon T cell activation, c-Rel, a component of the NF-κB signaling cascade, acts as a transcriptional activator of *Ezh2* expression (64). However, c-Rel was not required for resting T cell *Ezh2* expression, leaving the possibility for alternative transcriptional activators of *Ezh2* (64). EZH2 function is also determined by the natural turnover of the markers; thus, the kinetics of cell division play a factor in EZH2's role in forming the CD8+ T cell epigenetic landscape. Initial T cell activation is known to trigger EZH2 expression, however, whether this induction occurs to a similar extent in cases of chronic antigen stimulation like cancer has not been explored (28). In a similar vein is the interacting partners of EZH2 which could alter during different proliferative events such as memory reactivation vs. reinvigorating stem-like populations in cancer and chronic infection, which may proffer a means to target distinct T cell programs preferentially. Although many studies employ ablation models to study EZH2 function, this circumnavigates the complex question of how posttranslational modification of EZH2 via phosphorylation contributes to greater biological effects (2). Indeed, the use of EZH2 variants has demonstrated that phosphorylation status of EZH2 specifically in CD8+ T cells can impact antitumoral immunity (29). Thus, many open questions remain about the regulation, function, and role EZH2 plays in CD8+ T cell biology.

In this review, many studies are presented that demonstrate the differing effects of loss or pharmacological inhibition of EZH2 can exert on T cell activation, differentiation, and function. Despite common threads, there remain different results from many *in vivo* and *in vitro* models that warrant further investigation to fully explain what role EZH2 plays in CD8+ T cells. Furthermore, a more comprehensive understanding of the EZH2 network lends itself to further possible therapeutic avenues for the pursuit to augment or dampen CD8+ T cell responses.

## Author Contributions

CS, GT, and SS-A: manuscript drafting. All authors: directly provided contributions, read, and approved the final manuscript.

## Conflict of Interest

CS, GT, and SS-A were employed by Pfizer Inc. Pfizer employees may hold stock/stock options in the company.
